# Being a Newly Qualified Nurse: A Nordic Focus Group Study

**DOI:** 10.1177/23779608241244679

**Published:** 2024-04-01

**Authors:** Anette Tast, Anne Kasén, Karin Bölenius, Yvonne Hilli

**Affiliations:** 1 Faculty of Nursing and Health Sciences, 1786 Nord University, Bodø, Norway; 2 Department of Nursing, Umeå University, Umeå, Sweden

**Keywords:** Caring science, focus group interview, hermeneutics, newly qualified nurse, qualitative

## Abstract

**Introduction:**

The transition to working life as a newly qualified nurse (NQN) can be challenging, leading to heightened stress levels. While NQNs are generally enthusiastic about starting their careers, they often express concerns about various responsibilities and a perceived lack of experience in independently dealing with clinical care in complex environments.

**Objective:**

To acquire an in-depth understanding, from a caring science perspective, of what it means to be an NQN during the transition period of the first 18 months in the profession.

**Methods:**

This study relied on an exploratory qualitative design. The methodological approach followed Gadamer's hermeneutic philosophy. Six focus group interviews were conducted in northern Norway (n = 3) and northern Sweden (n = 3) from January through May 2021. The interpretation of the data was inspired by Fleming et al. Nineteen female and seven male NQNs working in different contexts, including hospitals and municipalities, participated in the study. The consolidated criteria for qualitative research were used to report the results.

**Results:**

Perspectives on NQNs are presented as three themes: a) *the responsibility was perceived as a significant challenge,* b) *being a nurse is complex and demanding,* and c) *a desire for personal and professional development.* Learning to be a nurse shouldering responsibility necessitates support and guidance from caring and compassionate colleagues and leaders.

**Conclusions:**

This study sheds light on the importance of creating a workplace culture where NQNs’ learning is promoted and supported by designated mentors during their transition to working life. The responsibilities should be aligned with their level of knowledge. It is important that leaders hold developmental dialogues and ensure a career plan for NQNs to continuously develop their knowledge and skills. Intervention studies designed to evaluate the meaning of the support from appointed mentors within structured mentorship programs are needed.

## Introduction

Being a newly qualified nurse (NQN) entering the workplace is challenging, leading many to experience elevated levels of stress (Duchscher & Windey, 2018; Rudman et al., 2020; Smythe & Carter, 2022; ten Hoeve et al., 2018). Among the reported factors are insufficient introduction and support from leaders and a lack of mentorship. Other contributing causes include heavy workloads and unsatisfied individual expectations (Çamveren et al., 2020). In this study, NQN refers to a registered nurse with no more than 18 months of work experience. This transition period is crucial for professionals; it might increase their commitment or trigger thoughts of leaving the profession (Masso et al., 2022). NQNs often face difficulties in reconciling their clinical practice ideals with the practical realm, leading to internal conflicts. Such conflicts are particularly relevant during the shift from being a student to becoming a nurse. The work environment can differ vastly from what is expected (Duchscher & Windey, 2018; Hawkins et al., 2019), leading to emotional shock. This phenomenon is supported by recent studies (Aldosari et al., 2021; Duchscher & Windey, 2018; Serafin et al., 2020; Smythe & Carter, 2022).

## Review of Literature

The initial period in nursing is often characterized by ambivalent feelings, including insecurity and anxiety about responsibilities or making mistakes. On the other hand, the initial period may offer a sense of satisfaction with the nurses’ ability to apply their knowledge and function independently. Developing a good relationship with patients and making a difference can be motivating factors. NQNs express a desire to learn and an awareness that the profession involves lifelong learning and the necessity to maintain their knowledge continuously (Bjerkvik et al., 2022; Hawkins et al., 2019; Serafin et al., 2020; ten Hoeve et al., 2018).

Most NQNs feel unprepared to work independently in the complex clinical reality (Aldosari et al., 2021; Ho et al., 2021; Najafi & Nasiri, 2023; ten Hoeve et al., 2018). Many NQNs have expressed that they lacked the opportunity, as a student, to acquire a broad experience of the nurse's role in the profession and to manage comprehensive responsibility (Bjerkvik et al., 2022; Hawkins et al., 2019; Ho et al., 2021; Smythe & Carter, 2022). Several studies report high degrees of stress among NQNs due to their responsibility in the new role (Aldosari et al., 2021; Ho et al., 2021; Rudman et al., 2020; Smythe & Carter, 2022; Sterner et al., 2018). A study by Bjerkvik et al. (2022) found that NQNs had significant responsibilities early in their jobs, with 90% entrusted with multiple patients within their first month of employment. Other studies have concluded that nurses had to assume more responsibility than they felt ready for (ten Hoeve et al., 2018; Walker et al., 2017; Willman et al., 2021).

If NQNs do not receive sufficient support, they are more likely to feel overwhelmed and insecure (Graf et al., 2020). A heavy workload, complex situations, and acute events leave little time to consolidate previous learning. Organizing work and making clinical judgments can be challenging, especially with an inexperienced team of nurses (Aldosari et al., 2021; Bjerkvik et al., 2022; Sterner et al., 2018). Challenges faced by NQNs include taking charge, leading teams, and communicating with doctors, patients, and relatives. Caring for patients with severe illness or at the end of life was also reported as an area where NQNs felt unprepared. These situations triggered feelings of hopelessness and emotional stress. Sharing feelings with colleagues and leaders helped NQNs face challenging situations (Bjerkvik et al., 2022; Masso et al., 2022; ten Hoeve et al., 2018).

A review of reviews concluded that NQNs lack self-confidence during their initial months in practice. However, self-confidence gradually increased with prolonged exposure and more experience (Masso et al., 2022). Earlier studies suggest that providing necessary support to nurses within the first six months improves skillsets and self-confidence (Hawkins et al., 2019; Masso et al., 2022; Smythe & Carter, 2022). Such development can be facilitated by mentorship or introductory programs, good relationships in the work environment and constructive feedback from experienced nurses, colleagues and patients. Experiences that inhibit self-confidence can be related to poor communication and inconsistency between theory and practice (Ho et al., 2021; Najafi & Nasiri, 2023; ten Hoeve et al., 2018).

The ability to support colleagues was also perceived as critical and provided a sense of satisfaction (Ho et al., 2021; Hoeve et al., 2018). When NQN's values align with those of their colleagues, feelings of belonging and engagement occur. If the values conflict with workplace values, such as not providing support to each other with the workload, or being unable to provide adequate care, or lack of emotional support from leaders to teams following particular incidents or if NQNs feel like they do not fit in, it might cause discomfort and a desire to leave the job (Ho et al., 2021; Smythe & Carter, 2022). A sense of belonging within a supportive team motivated staying in the workplace (Hawkins et al., 2019; Masso et al., 2022; ten Hoeve et al., 2018).

Negative experiences were common, such as a lack of support from colleagues and leaders or feeling undervalued (Çamveren et al., 2020; Hawkins et al., 2019). Increased responsibility without support can contribute to feelings of loneliness, uncertainty, insecurity and alienation (Ho et al., 2021). Being subjected to unprofessional treatment and a lack of solidarity in the workplace is a common experience (Çamveren et al., 2020; Hawkins et al., 2019; Masso et al., 2022; Najafi & Nasiri, 2023). NQNs who decided to leave the workplace were often left to handle challenges on their own. Under such conditions, they felt excluded, ignored and poorly treated (Çamveren et al., 2020; Ho et al., 2021).

The transition to the professional world is a complex and multifaceted process that appears to be influenced by workplace culture in terms of how NQNs perceive and navigate this initial phase (Çamveren et al., 2020; Hawkins et al., 2019; Masso et al., 2022; Smythe & Carter, 2022). The literature review shows that many quantitative studies were conducted in different hospital settings, with most of them focusing on skills and abilities. However, there is a shortage of qualitative research in this area, with a focus on being a NQN, and only a few studies adopting a caring science perspective. Therefore, this study aims to explore the topic from a caring science perspective using a qualitative methodology and acquire an in-depth understanding of what it means to be an NQN when entering the profession during the transition period (1–18 months).

The theoretical perspective is grounded in the tradition of caring science, which embraces an ethos of love, compassion and respect for human dignity. The ethos is embedded in the essence of human beings and serves as a motivating force (Eriksson, 2018). The ethos of NQNs becomes evident in their manner of conduct and in the atmosphere of the work environment. Although each NQN is unique, they long for a sense of fellowship and belonging to the prevailing culture. When they can act according to their values, they feel a sense of belonging or metaphorically at home (Hilli & Eriksson, 2019). Learning is a continuous process that involves personal and professional growth and development, moving continuously between the actual and the potential states, that is, ‘becoming’ (Gadamer, 2013; Sandvik & Hilli, 2022). According to Gadamer (2013), this process of inner formation continues throughout life.

## Methods

### Design

This study has an exploratory qualitative design. The methodological approach follows Gadamer's philosophical hermeneutics (Gadamer, 2013) to achieve an in depth understanding of what it means to be an NQN.

The authors pre-understanding includes the theoretical perspective, previous research, and the clinical and pedagogical experiences. A new understanding is made possible by reflecting on and challenging the pre-understanding. Thus, the fundamental prejudices of the tradition become a condition for understanding, made possible by a dialectical process with the interpreted text, initiated by something foreign that ‘speaks to us’. Through open dialogue with the text, our pre-understanding meets the text's horizon, thus revealing the phenomenon through a fusion of horizons. The interpretation process is characterized by a movement between parts and the whole and between our pre-understanding and understanding of the text in its entirety: the hermeneutic circle (Gadamer, 2013).

#### Setting and Inclusion Criteria

This study is part of a Nordic collaborative research project, between two universities, hospitals and municipalities in northern Norway and northern Sweden. The study recruited a convenient sample from the project partners in hospitals and municipalities. The leaders were provided with oral and written information regarding the study purpose and the inclusion criteria. They forwarded the information to potential participants. The eligibility criteria included: 1) NQNs with work experience ranging from one to 18 months, 2) currently employed for at least 75%, and 3) the ability to communicate in Norwegian or Swedish. The demographic information of the eligible participants was collected by facilitators, who were contacted by e-mail and telephone.

### Focus Group Interviews

Focus group interviews were used because they are useful for capturing the participants’ experiences and understanding the phenomenon under investigation (Kitzinger, 2005). Six digital focus group interviews were conducted during working hours between January and May 2021 – three in each country. The number of participants in the focus groups ranged from three to seven per group, with an experienced and a junior researcher participating in the interviews in each country (Krueger et al., 2015; Willemsen et al., 2023). The interviews had an open and conversational character, a dialogue in which all the participants were encouraged to share experiences. An interview guide ensured equivalence in all the focus group interviews. The interview began with an open-ended question: “Can you please tell us what it means to be an NQN?” The participants were encouraged to develop their thoughts on the theme with follow-up questions, such as: “Can you expand on your thoughts?” The participants were motivated and actively engaged in the conversation, resulting in a rich data source. The interviews were conducted using Microsoft Teams™ or ZOOM™. Field notes were taken simultaneously. The interviews lasted between 59 and 106 min and were recorded and transcribed.

### Data Analysis

The textual interpretation was inspired by Fleming et al. (2003), who described the interpretation process in four steps concerning Gadamer's hermeneutic philosophy (2013). The pre-understanding was documented by note taking throughout the process. The data were explored with an open mind by letting the text speak for itself to discover different nuances. As a first step, the interpretation process began with reading the entire data, followed by a meeting of the research group to reflect on the preliminary understanding. The first author was responsible for the analysis and continued the process by carefully reading all the data several times to capture the overall meaning of the texts. In the second step, each sentence and paragraph were scrutinized to discover and reveal several aspects of what it means to be an NQN, and units of meaning were identified. This phase provided a rich and extended understanding of the phenomenon ‘Die Sache’ (Gadamer, 2013). Themes and sub-themes were abstracted by asking: What is this about in a deep sense? In the third step, the different themes were continuously reflected against the pre-understanding, distinct parts and the whole (Table 1). Throughout the process, there was continuous dialogue with the data in the form of questions and answers, enabling a dialectical movement. In the interpretation process, the distinct parts were related to the text as a whole and back again to uncover an extended and deeper understanding. The data were continually interpreted with senior researchers, that is all authors. In the final step, we reflected on the text, challenging and expanding our pre-understanding. This led to a fusion of horizons and the emergence of a new, deeper, and shared understanding.

**Table 1.** Units of Meaning, Sub-Themes, and Main Themes.

Units of meaning	Sub-Themes	Main themes
A profound change to have an independent responsibility	*The realization of responsibility*	The responsibility was perceived as a significant challenge
A significant change, a shock, overwhelming feelings, and stress	*Entering the clinical workplace with personal responsibility*	
Inexperienced, fear of unforeseen situations and making mistakes, feelings of worry and stress	*Feelings of insecurity and uncertainty*	
Difficult to assess what is reasonable, challenging to make decisions and stand up for them	*A wide range of healthcare-related demands arise in a fast-paced work environment*	Being a nurse is complex and demanding
Difficult to communicate in sensitive situations, and to take and respond to criticism, difficult to work in a team, and not feel ready to lead the team	*Communication and collaboration within the team regarding patients, and relatives*	
Feelings of not being good enough, feeling inadequate, feelings of being left out, not feeling ready for a supervisory or teaching role	*Feelings of inadequacy and abandonment*	
The patient contact provides good motivation, a helping role, and learning new things both personally and professionally	*The motivation for learning and developing as a nurse*	A desire for personal and professional development
A good working environment, a welcoming atmosphere, and a safe space for learning allow one to be new and gain experience over time	*A caring work environment provides time and space to be new*	
Feeling welcome, inclusion, and belonging, to be seen and heard by the leader, being cared for, want to be involved and contribute to a good atmosphere and a learning culture	*A sense of belonging and being cared for*	

### Ethical Considerations

Approval for the study was granted by the Norwegian Agency for Shared Services in Education and Research, Trondheim (No. 148896) on December 18, 2020, and the Swedish Ethical Review Authority, Uppsala (Dnr 2020-06187) on March 03, 2021.

Participants, not known to the interviewers were given written and oral information on the study's purpose and provided written consent, with awareness of their right to withdraw at any point. Confidentiality was emphasized before the interviews, with the group-level presentation of results ensuring individual anonymity. The dataset was anonymized during transcription. Six focus group interviews were coded as NO.1–3 and SWE.1–3.

## Results

### Participant Characteristics

Forty NQNs were invited to participate in the focus group interviews. Three of them declined, while 11 were unable to join due to a high workload, the COVID-19 pandemic, or other reasons. A total of 26 NQNs took part, with 19 being women and seven being men. Their ages ranged between 22 and 42 years of age. The participants’ work experience spanned from three to 16 months, and most (n = 24) held full-time positions. The majority (n = 20) worked in specialist care in a variety of contexts, such as medical, surgical, paediatric, oncological, psychiatric, geriatric and rehabilitation units. Additionally, six participants were employed in municipal primary healthcare, residential or rehabilitation units, nursing homes or in-home care.

### Being a Newly Qualified Nurse

A pattern emerges in the results where the responsibility of NQNs permeates all themes. The study presents the results in three main themes with sub-themes: a) *responsibility was perceived as a significant challenge,* b) *being a nurse is complex and demanding,* and c) *a desire for personal and professional development* (Tabel 1)*.* The themes should not be viewed chronologically, but as parts that deepen and broaden the understanding of a complex whole: the meaning of being an NQN in a complex work environment.

#### Responsibility was Perceived as a Significant Challenge

The personal responsibility of an NQN was perceived as overwhelming and a heavy burden. There was a profound shift in responsibility from being a student, which was both challenging and exciting. Many found the extent of responsibility shocking when they entered the work environment. NQNs aim to gradually take on more responsibility with the guidance and support of experienced nurses.

**The realization of responsibility** was prominent even among those NQNs familiar with the workplace from previous clinical training in the unit. The need to make decisions about patient care and to trust their observations, assessments and decisions was a profound change. This feeling was both challenging and exciting: “It's a big step up from before you received your qualification … although it is a small step, it still feels so big because you must do everything yourself … it is a huge responsibility” (SWE.3). The newly acquired responsibilities were perceived as challenging but also motivating and providing an opportunity to demonstrate their acquired knowledge: “… exciting to show everything you've learnt at school” (NO.1).

**Entering the clinical workplace with personal responsibility** was perceived as a major change from being a student, and for many, “…it was a bit of a shock…” (NO.2), an unexpected and overwhelming experience. Becoming familiar with the routines of the work environment, colleagues, culture and the amount of new information to learn required a lot of energy. The initial year was regarded as a transformative phase of upheaval: “…there is something about that first year. There's so much to take in – it's overwhelming, that first year…” (NO.1).

Several NQNs experienced inadequate induction into the workplace: “There wasn’t much of an introduction – perhaps because they were familiar with us from before and we already had some idea of what working there as a nurse would involve” (NO.3). The heavy workload, fast pace, and time constraints contributed to increased stress levels and a perceived lack of resources to learn and consolidate all the new information. A shortage of experienced nurses in various workplaces sometimes results in insufficient support. Consequently, tasks were assigned without proper follow-up on their skills and knowledge.

NQNs expressed **feelings of insecurity and uncertainty** in caring for patients and working independently. They were concerned about facing unforeseen situations and making mistakes. At the end of their shifts, they often worried about whether they had provided adequate care for their patients. Furthermore, some were obligated to work as charge nurses, resulting in increased worry, insecurity and stress since they had to take on the same level of responsibility as experienced nurses: “We have the same level of responsibility as nurses who have been working for ten years. I felt quite stressed about this in the beginning” (SWE.3).

The NQNs wanted the opportunity to take on responsibilities and challenges within the scope of their competencies and to find their way of being nurses. They showed this by taking responsibility, being willing to explore new approaches, expanding their comfort zones by facing uncomfortable situations and seeking support when needed. They hoped for a work environment that allowed them to gradually take on more responsibility, developing their professional skills and understanding, supported by experienced nurses.

… that you are trusted, but at the same time, that you remain humble. … Yes, you are willing to learn new things, to listen to the people around you to learn from them … to find your way of doing things… (NO.2)

#### Being a Nurse is Complex and Demanding

Being an NQN in a fast-paced and complex environment can be a multifaceted and demanding experience, requiring organizing skills, collaborative leadership, multitasking, communication and teamwork with patients, relatives and inter-professional colleagues. A deep commitment was shown, but assessing patients, addressing complex situations and considering the needs of individual care were demanding.

**A wide range of healthcare-related demands arise in a fast-paced work environment**. Being responsible for and performing a variety of healthcare procedures and handling unfamiliar medical equipment was demanding: “It felt like being thrown into the thick of the action. We encountered many procedures which we had only seen once, two years ago [laughs]” (NO.3). Additionally, a sense of disorder was reported when being responsible for multiple patients, duties, and medication administration within time constraints: “…it can be hard to determine what is reasonable. It can vary so much from patient to patient. … It can be difficult, especially when they need something fast. You must be sure that it's a safe and reasonable dose” (SWE.1).

NQNs expressed dedication to providing attentive patient care and making clinically and ethically sound decisions. Assessing and prioritizing patients’ care needs and situations was perceived as demanding due to their limited clinical experience. Seeing and considering different perspectives was challenging, especially in acute and complex situations. In particular, pediatric, geriatric and palliative care patients and those with multiple illnesses posed challenges.

…this thing about the clinical gaze … that we need to see the whole patient. … We must make our own decisions and then stand by the decisions we’ve made. It's your view of the situation that can pick up if a patient is in a critical situation, so it's your observations that are crucial to getting them the help they need … that you make good decisions that are ethically correct… (NO.2)

**Communication and collaboration within the team regarding patients and relatives** were viewed as demanding. Many NQNs lacked prior experience in end-of-life care and found it demanding to face patients and families in crises or other sensitive situations. Communicating with patients suffering from dementia or those with aggressive behavior presented a challenge. Moreover, they struggled to converse with relatives amidst a busy schedule and were criticized by patients, relatives or doctors: “…we haven't built up the experience or the competence to stand in the room of a dying patient in the company of relatives … to be in front of them and take their conversations and their criticisms…” (NO.3).

Although teamwork was a valuable learning experience, collaboration with patients, families, fellow healthcare professionals and healthcare units for the best interests of the patient was found to be demanding: “… how to work together so that the patient is okay when they go home…” (NO.2). The NQNs experienced challenges in self-management that affected their ability to be team workers and team leaders. Assuming this new role was demanding and perceived as inappropriate. However, recognizing their limitations led them to turn to nursing assistants for support. The absence of an experienced registered nurse for reflection and guidance posed an additional difficulty: “It has been good to have the nurse assistants to ask questions and discuss things, but I’ve missed having a nurse to discuss things with…” (NO.1).

**Feelings of inadequacy and abandonment** were common when encountering unfamiliar situations and lacking sufficient knowledge to solve problems or complete tasks. A heavy workload, time pressure and a fast pace could cause stress and emotional strain for NQNs, particularly if they could not provide high-quality patient care. Some perceived themselves as a burden to their colleagues.

It can feel like you're not good enough, and then you think: Should I know all this? … Eh, you have these feelings of inadequacy … you want so badly to have time to do everything … you become a burden to the nurse who comes after you. (SWE.3)

When the demands of responsibility became too overwhelming to handle alone, particularly in complex care situations, it sometimes resulted in feelings of being abandoned. This led to isolation and emotional distress, making it challenging to sustain the motivation to continue working.

… for someone with basic training to have critically ill patients … um, you felt afraid to go to work … as there is a lot of responsibility on you when you don't have … I don't know how to explain it. But yes, I think it's hard. (SWE.2)

Teaching students and introducing new staff to the workplace presented significant challenges and felt too demanding. NQNs felt unprepared to act as a preceptor or have an introductory role during their learning process: “… I feel that I haven't acquired sufficient knowledge to train another person … I wasn’t even ready myself … I’m not yet confident enough in my role to respond to questions about how things work …” (NO.1).

#### A Desire for Personal and Professional Development

NQNs expressed motivation and a desire to learn and develop as nurses. They seemed aware of their limitations and approached their work with humility. They called for a workplace culture that enabled them to learn and develop as caring nurses with the support of experienced colleagues and leaders. A sense of belonging contributed to greater job satisfaction and reduced feelings of isolation or abandonment.

**The motivation for learning and developing as a nurse**, both personally and professionally, was expressed. The new professional role entailed great enthusiasm and anticipation: “… you have the basic knowledge and can then use your personal qualities to build upon that” (NO.3). NQNs expressed a genuine interest in patients and a motivation to care for others. They had a holistic view and expressed a desire to be caring nurses. Actively engaging in patient care was seen as fulfilling and rewarding: “… one of the most important things in the work of a nurse and a good motivator is patient contact, which is very rewarding … that you see the whole patient and focus on each individual…” (NO.2).

NQNs appear dedicated to enhancing their confidence and autonomy in caring responsibilities, mastering their profession and identifying with their professional title. They found their work meaningful and multifaceted, with many learning opportunities. Positive feedback from patients and rapid progress in learning gave them a sense of satisfaction. Experienced nurses with a caring approach served as role models.

… to feel confident in the role you have as a nurse and the responsibility you have … I look up to those who are competent, and hope that I can one day be as good as them … they see or do that little bit extra… (NO.2)

**A caring work environment provides the time and space to be new.** The caring attitude among colleagues, understanding that they were new and needed time to learn to be nurses, provided a safe space for learning. An atmosphere where support was offered promoted development and gave a sense of security in the learning process: “That's when we must start learning, as everyone had said … they don’t mind being asked stupid questions. They told us it was okay to be new and said, feel free to come and ask me” (NO.3).

When NQNs received support from personal mentors, their initial period was perceived as an educational, positively challenging, and a satisfying process of continuous development: “… it's been very rewarding, and I’ve always had someone to ask – and I was assigned a mentor early on … for me, being new has been fun and educational” (SWE.1).

NQNs emphasize the importance of personal mentorship and collegial support for successful development as a nurse. In a caring and learning culture, there are opportunities to discuss and reflect on routines, challenges, demanding situations, errors, and misjudgments. Through experience gained over time, supported by mentors and colleagues, they were allowed to progress in their personal and professional development: “…it has taken a long time for me to feel that I am becoming independent…” (SWE.2).

**A sense of belonging and being cared for **was expressed by NQNs as feeling welcome and included in the work environment: “…the atmosphere in a workplace and the relationship with colleagues, that is perhaps something that I value very highly if not most highly” (SWE.1). Such an atmosphere gave a sense of fellowship and belonging. In addition to relationships with colleagues, the importance of the leader was emphasized. Only a few NQNs had the opportunity to have dialogues with their leaders and discuss challenges and their professional development. Being seen, heard, acknowledged, and supported by the leader gave them a sense of being appreciated and cared for: “We had a meeting, where we were free to bring up the feelings that we, the new ones, had – yes, we are well taken care of” (NO.3). NQNs called for dialogues with leaders to clarify expectations, responsibilities and career advancement possibilities.

Supporting colleagues and working as a team gave feelings of inclusion and belonging, fostering unity, job satisfaction and, ultimately, well-being. A workplace culture that promoted learning and development inspired continuous learning and was desirable.

That you are a good colleague, and that you are involved and contribute to a good atmosphere … that you ask whether there's anything you can help with and that you contribute to such a culture. … Being involved and pushing through change and promoting new research and actively participating in it… (SWE.3)

## Discussion

This study aimed to acquire an in depth understanding of what it means to be an NQN during the transition period, guided by Gadamer's hermeneutics (2013). The discussion reflects the results in light of the theoretical perspective and previous studies.

Our study showed that being an NQN shouldering a great responsibility was experienced as both challenging and exciting. Above all, it involved a profound and transformative change where the horizon, a somewhat idealized picture of nurses’ work, encountered the horizon of a hectic work environment (Hawkins et al., 2019). The overwhelming experience in the results has also been confirmed in previous studies (Aldosari et al., 2021; Duchscher & Windey, 2018; Smythe & Carter, 2022). Awareness is a turning point when responsibility becomes personal and touches the inner ethical dimension. When something touches us or ‘speaks to us’, it is a starting point for a change that will be visible in actions (Gadamer, 2013). The moment when the nurse sees, realizes and becomes aware of the personal responsibility in the professional role can be likened to a defining moment when the understanding is expanded (Duchscher & Windey, 2018; Eriksson, 2018; Gadamer, 2013; Sandvik & Hilli, 2022).

The core values, ethos, are expressed by NQNs in this study as a deep sense of responsibility, commitment, and a desire to learn and be caring nurses. These values constitute an inner ethical dimension – a driving force that becomes evident in the manner of being and actions (Hilli & Eriksson, 2019). Previous studies have shown that most NQNs do not feel ready to shoulder great responsibility alone (Sterner et al., 2018; ten Hoeve et al., 2018; Walker et al., 2017) or to work independently (Aldosari et al., 2021; Hawkins et al., 2019), which is also supported by this study. In our study, NQNs said they had to face an excessive level of responsibility at the beginning of their careers by being exposed to challenging situations with insufficient preparation, which could result in high-stress levels (Bjerkvik et al., 2022; Masso et al., 2022; Sterner et al., 2018; ten Hoeve et al., 2018; Willman et al., 2021) while adequate support reduced stress (Ho et al., 2021).

This study concluded that taking on new challenges in a professional role was both inspiring and exciting. The clinical view was inexperienced, leading to a lack of confidence in clinical decision-making, as supported by previous research (Najafi & Nasiri, 2023; Walker et al., 2017). Over time, the NQNs in our study increased their confidence as they gained more experience, a finding supported by other studies (Masso et al., 2022). Expanded knowledge through experience is pieced together, forming a new understanding and horizon (Gadamer, 2013; Sandvik & Hilli, 2022). In this study, the NQN showed genuine dedication when dealing with demanding situations involving patients, relatives and other healthcare professionals. These situations could evoke feelings of inadequacy and a lack of control. Nevertheless, the ethical perspective serves as a guide, emphasizing the importance of recognizing limitations and embracing different perspectives. NQNs were learning to be and act as nurses, i.e., a process of becoming (Dall'Alba, 2009; Sandvik & Hilli, 2022) or formation. Formation encompasses more than the acquisition of skills, techniques and strategies (Bildung) (Gadamer, 2013).

When NQNs’ ambitions conflict with external expectations and demands, they may encounter an internal dilemma. According to our results, NQNs strived to balance external demands and internal resources. Feelings of inadequacy and isolation can lead to demotivation and a desire to switch employment and may affect overall well-being. Previous studies have described the incongruence between the expectations of working life and the level of competence of NQNs (Bjerkvik et al., 2022; Masso et al., 2022). Rudman et al. (2020) suggests that taking preventive actions early in NQN's career can help avoid excessive stress.

In this study, NQNs advocated a work environment in which they were given the time and space to be new and develop personally and professionally. A sense of belonging and being welcomed as a team member—being cared for—was highlighted as particularly important. In a welcoming atmosphere in which nurses are welcomed and supported by colleagues and leaders, the experience of shock can be reduced or even eliminated (Ho et al., 2021). The formative process does not occur in isolation, but together with and in fellowship with other actors in a nurturing culture (Gadamer, 2013). This can be related to the metaphor of the ‘home’ and its reciprocity. The culture of the work environment is created by its inhabitants, and the inhabitants are formed in the culture. The formation is a dynamic process in which NQNs feel a sense of belonging and at home (Aldosari et al., 2021; Hawkins et al., 2019; Hilli & Eriksson, 2019; Sandvik & Hilli, 2022).

The importance of workplace culture has received little attention in previous studies, while this study highlights its importance. Earlier studies suggest that mentorship programs are a source of support for NQN during the transition period. However, the availability of mentors, their pedagogical skills and the relationship between the mentor and the mentee are critical (Masso et al., 2022). In this study, the NQNs emphasized the importance of having an appointed personal mentor. However, it became evident that the lack of a personal mentor could be compensated if the unit's culture was permeated by supportive colleagues who embraced them. This is consistent with earlier studies showing that a positive and supportive workplace culture helps to promote transition (Masso et al., 2022; Smythe & Carter, 2022). The absence of a positive culture has the opposite effect, particularly by harming self-confidence.

## Strengths and Limitations

The study's strength is that the participants represented different contexts in hospitals and municipalities and were from two Nordic countries, offering a rich and diverse picture. The open-ended nature of the research questions enabled rich data, which enabled a more profound understanding, consistent with Fleming et al. (2003). The trustworthiness of the study is strengthened by the participation of both female and male nurses of different ages. A limitation may be that few male nurses were represented and thus some perspectives may have been lost. On the other hand, the number of men exceeded their representation within the profession. COVID-19 affected data collection due to a high workload and absence from work during the pandemic. As a result, several invited participants did not attend, meaning that some focus groups had few participants. Under these conditions, all interviews were conducted digitally. This may have affected the interaction in the conversations due to unfamiliarity with communicating via screen, difficulties in reading body language, and challenges with the technical equipment. Several workplaces had insufficient computer equipment and facilities for participating in an interview, which might have affected the results and is therefore a limitation. Conducting focus group interviews via digital platforms is relatively new, and knowledge is limited (Willemsen et al., 2023). However, the interviews provided rich data, and despite these limitations, the results can be cautiously transferred to other contexts.

## Implications for Practice

This study shows the importance of facilitating structured mentorship programs with designated mentors to introduce and support NQNs in developing confidence in their roles and responsibilities. It is essential to create a learning workplace culture that promotes a sense of belonging and ensures well-being in the workplace. Leaders are pivotal in cultivating a learning culture and extending the necessary support. Attention should focus on creating and developing mentorship programs and frameworks for career advancement.

## Conclusions

This study sheds light on the importance of creating a workplace culture where learning by NQNs is promoted and supported. Given their individual needs, they should receive adequate and structured introductions and support from designated mentors during their transition to working life. The tasks and responsibilities should be aligned with their level of knowledge. To ease the transition from student to NQN, it is recommended to take on responsibilities of being nurse progressively. The importance of leaders holding developmental dialogues and ensure a career plan for NQNs to continuously develop their knowledge and skills was emphasized in this study. Further research is essential to explore the meaning of supportive and learning work environments for NQNs. Intervention studies designed to evaluate the meaning of the support from appointed mentors within structured mentorship programs are needed.
